# Morin hydrate downregulates inflammation‐mediated nitric oxide overproduction and potentiates antioxidant mechanism against anticancer drug doxorubicin oxidative hepatorenal toxicity in rats

**DOI:** 10.22038/AJP.2023.22392

**Published:** 2023

**Authors:** Ademola C. Famurewa, Chima A Ekeleme-Egedigwe, Patience N Ogbu, Ayodeji J Ajibare, Moshood A Folawiyo, Doris O Obasi, Arunaksharan Narayanankutty

**Affiliations:** 1 *Department of Medical Biochemistry, Faculty of Basic Medical Sciences, College of Medical Sciences, Alex Ekwueme Federal University, Ndufu-Alike, Ikwo, Ebonyi State, Nigeria*; 2 *Department of Pharmacology, Manipal College of Pharmaceutical Sciences, Manipal Academy of Higher Education, Manipal University, Karnataka State, India*; 3 *Department of Biochemistry, Faculty of Biological Sciences, Alex Ekwueme Federal University, Ndufu-Alike Ikwo, Abakaliki, Nigeria*; 4 *Department of Physiology, Faculty of Basic Medical and Health Sciences, College of Medicine, Lead City University, Ibadan, Oyo State, Nigeria*; 5 *Department of Physiology, Faculty of Basic Medical Sciences, College of Medicine, Ekiti State University, Ado-Ekiti, Nigeria*; 6 *Division of Cell and Molecular Biology, PG and Research Department of Zoology, St Joseph’s College (Autonomous), Devagiri, Kerala, India*

**Keywords:** Morin, Toxicity, Oxidative stress, Chemotherapy, Anticancer drugs, Flavonoids

## Abstract

**Objective:**

Doxorubicin (DOX) is a frontline antineoplastic drug that kills cancer cells through genotoxic mechanism; however, it induces organ toxicities. This study assayed whether morin hydrate (MOH) could abrogate DOX hepatorenal toxicity in rats.

**Materials and Methods:**

There were 4 groups of rats: Control, MOH, DOX and MOH + DOX. Rats were administered MOH (orally, 100 mg/kg bw) for 7 consecutive days, while DOX was injected (40 mg/kg, ip) on the 5th day only. Hepatorenal function markers, and glutathione peroxidase (GPx), superoxide dismutase (SOD), and catalase (CAT) activities were estimated in both organs. Hepatorenal glutathione (GSH), malondialdehyde (MDA), and nitric oxide (NO) levels were estimated with histopathology.

**Results:**

DOX significantly (p<0.05) reduced antioxidant enzyme activities and GSH level, while NO and MDA levels increased (p<0.05) compared to the control. DOX prominently altered hepatorenal indices and induced histopathological alterations. MOH abrogated the DOX hepatorenal toxicity and alleviated the histological lesions in the liver and kidney.

**Conclusion:**

MOH restored the indices via antioxidant mechanism and downregulation of NO overproduction in rats.

## Introduction

**Figure 1:**
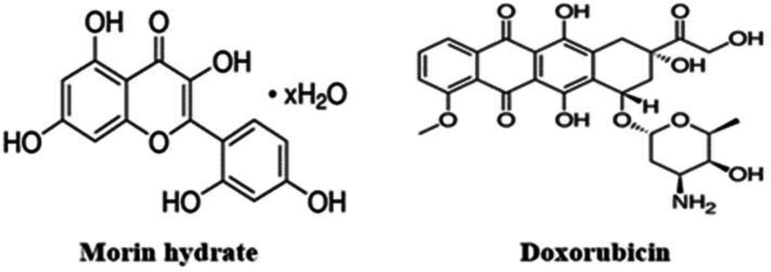
Molecular structure of morin hydrate and doxorubicin

Chemotherapy is a spearheading modality in anticancer treatment. However, the unpalatable adverse effects of chemotherapy are current hindrances to its clinical use, and sometimes chemotherapy reduces the patients’ quality of life (Zhang et al., 2018 ▶). Doxorubicin (DOX) is an anticancer drug used in various cancer therapies (Ashrafizadeh et al., 2020 ▶; El-Far et al., 2021 ▶). It is one of the leading efficacious anticancer drugs known as DNA intercalators (Thurston, 2007 ▶). The mechanism of action involves intercalation through insertion between the DNA base pairs. The amino sugar side chain of DOX (Figure 1) positions it in the DNA minor or major grooves blocking RNA transcription in cancer cells (Zhang et al., 2020 ▶; Thurston, 2007 ▶). However, DOX is associated with severe side effects and organ toxicity stemming from its stray action on healthy cells. Previous clinical and preclinical investigations show that DOX induces cardiotoxicity, hepatotoxicity and nephrotoxicity (Chatterjee et al., 2010 ▶; Hagag et al., 2020 ▶; Hussain et al., 2021 ▶; Liu et al., 2021 ▶; Thorn et al., 2011 ▶). In many animal models, DOX causes nephropathy associated with glomeruli destruction, tubule damage and filtration-barrier damage (Hekmat et al., 2021 ▶; Hussain et al., 2021 ▶). The DOX-induced renal damage is linked with significant increases in some biochemical indices, including serum urea, creatinine and uric acid. The study of Shivakumar et al. (2012) ▶ in rats indicates that DOX is a trigger of hepatocyte degeneration, bile duct hyperplasia and focal necrosis. The exact mechanism of DOX toxicity and damage is still unknown; however, the literature chiefly implicates DOX-induced oxidative inflammatory cascades (Benzer et al., 2018 ▶; Hussain et al., 2021 ▶; Sonowal et al., 2021 ▶). Doxorubicin provokes reactive oxygen species (ROS) generation and resultant deficit in redox balance mechanism (Hussain et al., 2021 ▶). It is known that the oxidative attack of ROS impairs integrity of biological tissues, including the liver and kidney. Accumulating evidence from animal studies show that DOX-induced ROS is the major promoter of toxicity, oxidative inflammation and aberrant signaling (Benzer et al., 2018 ▶). Thus, preventing DOX-mediated oxidative inflammation with antiradicals and anti-inflammatory compounds could abrogate DOX organ toxicity. 

The accumulating evidence on the efficacy of natural products against toxicities and pathologies is paving the way for their application in anticancer therapy (Khafaga et al., 2021 ▶). Natural products have potent antioxidant and anti-inflammatory capacities to protect tissues from oxidative damage (El-Far et al., 2019 ▶; Moghaddam et al., 2020 ▶). Morin (2΄,3΄,4,5,7-pentahydroxyflavonoid) (Figure 1) is a natural bioflavonoid compound present in vegetables, fruits, tea, mulberry figs and oriental medicinal herbs (Ahn et al., 2021 ▶, Sultana et al., 2017 ▶). Studies show its antioxidant, anti-inflammatory, antiangiogenic, antidiabetic, anti-hypertensive, anti-clastogenic, and cytoprotective activities in experimental models (Ahn et al., 2021 ▶; Fang et al., 2003 ▶; Sultana et al., 2017 ▶; Verma et al., 2019 ▶). Further, morin (MOH) attenuated toxicities of acrylamide and cisplatin, and effects of diabetic retinopathy via inhibition of oxidative stress, inflammation and apoptosis (Jiang et al., 2020 ▶; Kucukler et al., 2020 ▶; Singh et al., 2018 ▶). The study of Kok et al. (2000) ▶ reveals that MOH protects against anticancer drug toxicity without undercutting their anticancer efficacy. Therefore, the present study explored whether the antioxidant property of MOH could prevent DOX-induced hepatorenal toxicity in rats.

## Materials and Methods

**Drug and chemicals**

Morin hydrate (M4008) was bought from Merck Company, USA. Doxorubicin was obtained as DOX hydrochloride (Adriamycin PFS, 50 mg/25 ml injectable) from Khandelwal Laboratories Pvt. Ltd, Mumbai, India. It was purchased from a reputable Pharmacy Store in Abakaliki, Ebonyi State, Nigeria. Antioxidant enzymes were analyzed by chemical kits manufactured by Randox Laboratories Ltd., UK. Thiobarbituric acid (504176) from HiMedia Laboratories Pvt. Ltd, India was used for malondialdehyde analysis. Additional reagents used for this study were commercial products and of quality grades.

**Animals**

Wistar rats (male, 24 rats) that weighed 160–200 g were bought from a Rat Breeder in Abakaliki, Ebonyi State, Nigeria. The rats were subjected to 1 week of acclimatization. The standard conditions (22±1°C temperature; 50%‐65% relative humidity; 12‐hr light and dark cycle) were maintained for the rats. Commercial pelleted animal feed (Vital Feed, Jos, Nigeria) was served *ad libitum* to the rats throughout the experimental period. The study followed the approved protocols by Research and Ethics Committee of Ebonyi State University (EBSU/REC/BMS/1808/02/001). Also, animal experiment procedures of the National Institute of Health Guide for the Care and Use of Laboratory Animals (NIH Publications No. 80-23, revised in 1996) were followed. 

**Experimental design**

Before the study treatments, rats were randomly divided into four groups (n=6), and the following treatments were given to the rats: 

Group 1 (Normal control): Rats were given normal saline (2 ml/kg body weight (bw) for 7 days. 

Group 2 (MOH): Rats were given 100 mg/kg bw of MOH dissolved in normal saline, via oral gavage for 7 days (Ҫelik et al., 2020 ▶). 

Group 3 (DOX): Rats were given 40 mg/kg bw of DOX dissolved in normal saline, and intraperitoneally injected (ip) on the 5th day only (Benzer et al., 2018 ▶). 

Group 4 (MOH+DOX): Rats were given 100 mg/kg bw of MOH orally for 7 days +DOX (40 mg/kg, ip) on the 5th day only.

The doses of the drugs were chosen based on several studies performed and published previously (Celik et al., 2020 ▶; Benzer et al., 2018 ▶). The rats’ weight was recorded before and after treatment. Twenty-four hours after the final MOH administration, diethyl ether was used to anaesthesize the rats for blood collection. Samples of blood were withdrawn from the heart into non-anticoagulant tubes. All rats were sacrificed by cervical dislocation for removal of the kidney and liver tissues. The liver and kidney were cleansed with ice-cold saline water and their weights were determined. The blood samples were centrifuged (3500 g for 5 min) to obtain serum for indices related to renal and hepatic function. The tissues were separated for biochemical analyses and histology. Sample homogenization in phosphate buffered saline (PBS) (pH 6.4; 1:5 w/v) and centrifuged at 4000 g for 20 min (Haidari et al., 2019 ▶). The homogenate supernatant obtained was used for oxidative stress markers, lipid peroxidation and nitric oxide levels. The kidney and the liver samples were preserved in 10% buffered formalin for 48 hr before histopathological examinations (Omidifar et al., 2020 ▶).

**Analyses** **of** **biochemical indices**

**Estimation of hepatorenal function parameters**

Uric acid, urea and creatinine level were estimated for evaluation of kidney function. Albumin (ALB), total protein (TP), aspartate aminotransferase (AST), alanine aminotransferase (ALT), and alkaline phosphatase (ALP) were analyzed for evaluation of liver function. Commercial chemical kits from RANDOX were used following manufacturers’ instructions.

**Estimation of hepatorenal markers of oxidative stress**

The homogenate supernatant was used for oxidative stress markers. Catalase (CAT) activity was estimated based on the concentration of CAT that reduces H_2_O_2_ following Aebi (1983) method. Superoxide dismutase (SOD) activity was determined according to Marklund and Marklund (1974) using freshly prepared adrenaline solution. Glutathione peroxidase (GPx) activity was estimated based on method of Paglia and Valentine (1967) ▶ by reducing lipid hydrogen peroxide and hydroperoxides. The reduced glutathione (GSH) level was estimated by Beutler method (1975) consistent with the reduction of 5,5′-dithiobis-(2-nitrobenzoic acid) at 412 nm using UV spectrophotometer (UV-1650 PC, Shimadzu, Japan). The thiobarbituric reactive substance (TBARS) reacted with thiobarbituric acid and trichloroacetic acid for determination of malondialdehyde (MDA) content in both tissues following the method of Ohkawa et al. (1979) ▶. Green et al*.* (1982) method was used for analysis of nitric oxide (NO) level with the use of Griess reagent (0.2% N-(1-naphthyl) ethylenediamine dihydrochloride in deionized water and 5% sulfanilamide 2% in H_3_PO_4_). The absorbance of the chromogen pink azo dye was determined at 540 nm (UV spectrophotometer UV-1650 PC, Shimadzu, Japan).

**Histopathological examination**

The portions of tissues preserved in buffered formalin were used for histology. The ascending graded ethanol dehydration was carried out and the tissue was embedded in paraffin. The liver and kidney were sectioned and hematoxylin-eosin stained using routine methods for microscopic observation for histopathological alterations by Olympus CX41 microscope, Olympus, Tokyo, Japan (Omidifar et al., 2020 ▶). Kidney sections were graded for tubular necrosis, tubular degeneration/atrophy, and inflammation (Hekmat et al., 2021 ▶). For the liver, the severity of alterations was considered and scored based on necrosis, inflammation, degeneration, and congestion of portal vein (Özdemir et al., 2022 ▶). Three tissue sections from the liver or kidney were graded as showing: no histological damage (0), mild alterations (1), moderate pathological changes (2), and severe pathological changes with tubular degeneration (3) following extensive alterations according to their histopathological findings (Hekmat et al., 2021 ▶; Özdemir et al., 2022 ▶).

**Statistical analysis**

Data are presented as the mean±SEM, and results were analysed using one-way ANOVA (version 22; SPSS, Chicago, IL). *Post hoc* Tukey test was used to compare among the groups. Differences were considered significant at p<0.05.

## Results

**Effect of MOH and DOX on organs and body weight**

Table 1 depicts the influence of MOH and DOX on body weight and organ weight of rats. At the end of the experimental treatment, the weights of rats were similar (p>0.05). On the contrary, DOX injection markedly reduced the liver weight in comparison to the control (p<0.05), whereas MOH in the MOH+DOX group significantly increased the liver weight in comparison to the DOX group (p<0.05). However, MOH treatment had no significant effect on kidney weight (p>0.05)

**Effect of MOH and DOX on liver function markers**

Table 2 depicts the effect of MOH and DOX on serum markers of liver function in rats. The MOH in the MOH group exerted no effect (p>0.05) on the serum markers compared to the normal control. The DOX injection significantly increased the serum activities of AST, ALT and ALP, while TP and ALB levels decreased prominently in comparison to the normal control (All, p<0.05). On the contrary, MOH in the MOH+DOX group significantly decreased the enzyme activities and increased TP and ALB levels in comparison to the DOX group (All, p<0.05). 

**Effect of MOH and DOX on kidney function markers**

Table 3 reveals the effect of MOH and DOX on the markers of kidney function in rats. MOH in MOH group had no significant effect (p>0.05) on the serum markers in comparison to the normal control. The DOX injection significantly increased the serum levels of uric acid, creatinine, and urea in comparison to the normal control (p<0.05). On the contrary, MOH in MOH+DOX group significantly decreased the markers levels in comparison to the DOX group (p<0.05). 

**Table 1:** Effect of MOH and DOX on rat body weight, and liver and kidney weight

Group	Rat body weight (g)		Liver weight (g)	Kidney weight (g)
	Initial	Final		
Control	186.1±6.1	204.6±6.1	8.12±0.30	0.63±0.03
MOH	173.8±6.9	194.6±6.1	8.02±0.40	0.60±0.04
DOX	174.4±8.9	190.1±10.4	6.81±0.39*	0.57±0.02
MOH + DOX	180.3±6.3	200.9±7.6	8.13±0.40^#^	0.58±0.04

MOH: Morin hydrate; DOX: Doxorubicin; values are mean±SEM (6 rats/group). *p<0.05: significant difference compared to the control group in the same column; #p<0.05: significant difference compared to the DOX group in the same column.

**Table 2:** Effect of MOH on serum markers of liver function in DOX-administered rats

Group	ALT	AST	ALP	TP	ALB
Control	5.83±0.41	9.83±0.31	11.66±1.49	5.53±0.29	4.05±0.26
MOH	6.85±0.47	9.00±0.45	11.50±1.43	5.45±0.31	3.90±0.40
DOX	12.67±0.61*	21.80±0.94*	18.50±1.67*	1.97±0.27*	1.40±0.29*
MOH + DOX	7.00±0.51^#^	12.17±0.56^#^	12.16±0.47^#^	4.97±0.23^#^	3.85±0.24^#^

MOH: Morin hydrate; DOX: Doxorubicin; values are mean±SEM (6 rats/group). *p<0.05: significant difference compared to the control group in the same column. **#**p<0.05: significant difference compared to the DOX group in the same column.

**Table 3:** Effect of MOH and DOX on serum markers of kidney function in rats

Group	Creatinine	Urea	Uric Acid
Control	2.56±0.81	58.00±5.31	11.77±1.62
MOH	2.58±0.91	61.17±6.44	11.98±2.22
DOX	5.45±1.21*	120.84±9.40*	19.79±2.78*
MOH + DOX	2.78±0.81^#^	76.10±6.66^#^	10.26±2.10^#^

MOH: Morin hydrate; DOX: Doxorubicin; values are mean±SEM (6 rats/group). *p<0.05: significant difference compared to the control group in the same column. #p<0.05: significant difference compared to the DOX group in the same column.

**Effect of MOH and DOX on hepatic NO and oxidative stress markers**

**Figure 2:**
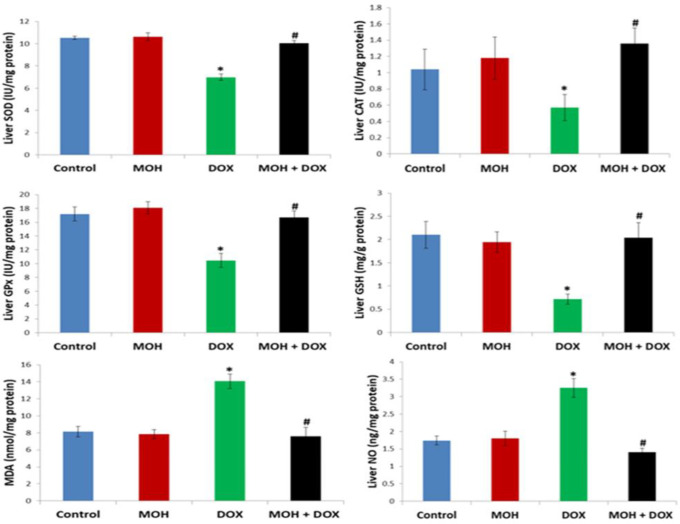
Effect of MOH and DOX on hepatic NO and oxidative stress markers. MOH: Morin hydrate; DOX: Doxorubicin; MDA: Malondialdehyde; GPx: glutathione peroxidase; SOD: superoxide dismutase; NO: Nitric oxide; CAT: Catalase; GSH: reduced form of glutathione. Values are expressed as mean±SEM (n=6). ^⁎^Significant difference compared to the normal control (p<0.05); ^#^significant difference compared to the DOX group

Figure 2 reveals the effect of MOH and DOX on hepatic activities of SOD, CAT, GSH and GPx, and levels of MDA and NO. Administration of 

DOX on the 5th day appreciably reduced the hepatic activities of SOD, CAT, GSH and GPx in comparison to the normal control (p<0.05). Also, DOX injection significantly increased levels of MDA and NO when compared to the normal control (p<0.05). On the contrary, MOH administered to rats in the MOH+DOX group prominently increased activities of SOD, CAT, GSH and GPx, whereas the levels of MDA and NO were reduced in comparison to the normal control group (p<0.05). 

**Effect of MOH and DOX on renal NO and oxidative stress markers**

**Figure 3:**
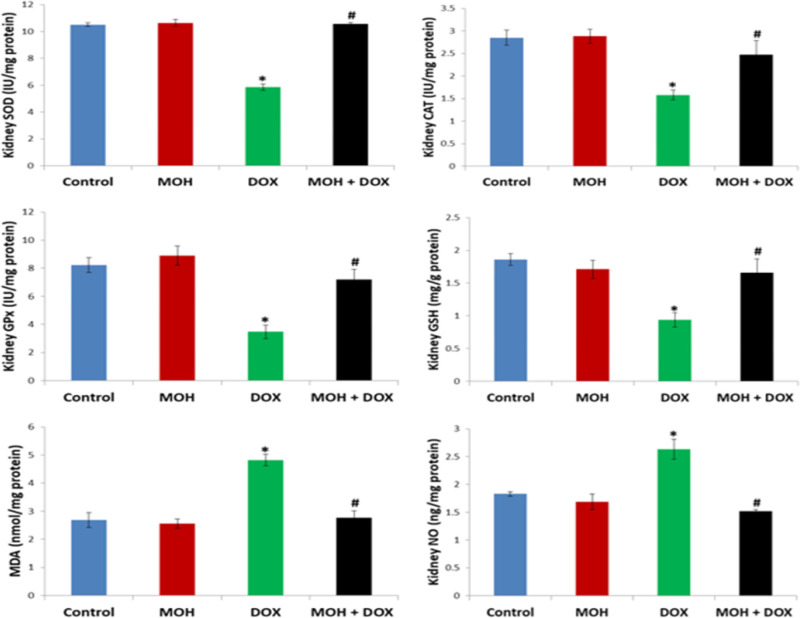
Effect of MOH and DOX on renal NO and oxidative stress markers. MOH: Morin hydrate; DOX: Doxorubicin; SOD: Superoxide dismutase; CAT: Catalase; MDA: Malondialdehyde; GPx: Glutathione peroxidase; NO: Nitric oxide; GSH: Reduced glutathione. Values are expressed as mean±SEM (n=6). ^⁎^Significant difference compared to the normal control (p<0.05); ^#^significant difference compared to the DOX group

Figure 3 presents the effect of MOH and DOX on renal activities of GSH, SOD, CAT, and GPx, and levels of MDA and NO. DOX injection on the 5th day markedly reduced the renal activities of SOD, CAT, GSH and GPx in comparison to the normal control (p<0.05). 

In addition, DOX injection significantly increased levels of MDA and NO in comparison to the normal control group (p<0.05). On the contrary, MOH administered to rats in the MOH+DOX group significantly increased activities of SOD, CAT, GSH and GPx, while the renal levels of MDA and NO were reduced in comparison to the normal control group (p<0.05).

**Effect of MOH on liver histology of DOX-injected rats**

**Figure 4:**
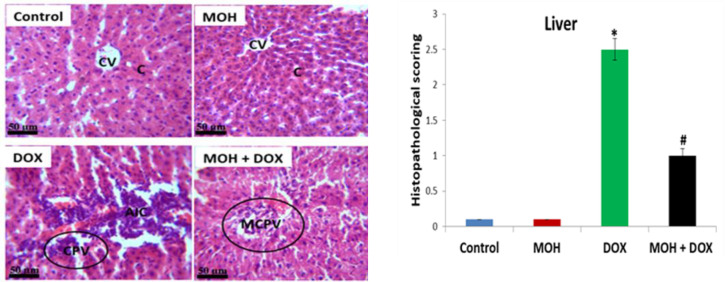
Photomicrographs of rat liver administered with MOH and DOX (H & E stain, 400×, n=3). Control group: Normal central vein (CV) and cytoplasm (C); MOH group: Normal central vein (CV) and cytoplasm (C); DOX group: Necrosis and aggregate of inflammatory cells (AIC) and congestion of portal vein (CPV); MOH+DOX: Mild congestion of portal vein (MCPV) with near normal appearance (p<0.05). *Significant difference compared to the normal control (p<0.05); **#**Significant difference compared to the DOX group

The examination of possible histopathological alterations was performed in this study (Figure 4). The cyto-architectural features of liver in control rats depict normal central vein and cytoplasm. The liver histology from the MOH control animals was consistent with that of the control animals (H&E × 400). On the contrary, the liver tissue from DOX-injected rats revealed severe lesions consistent with necrotizing cells, hemorrhagic abrasion and infiltration of inflammatory cells. In the MOH+DOX group, the MOH supplementation was found to alleviate the pathological effects seen in the DOX group such as mild congestion of portal vein.

**Effect of MOH on kidney histology of DOX-injected rats**

**Figure 5:**
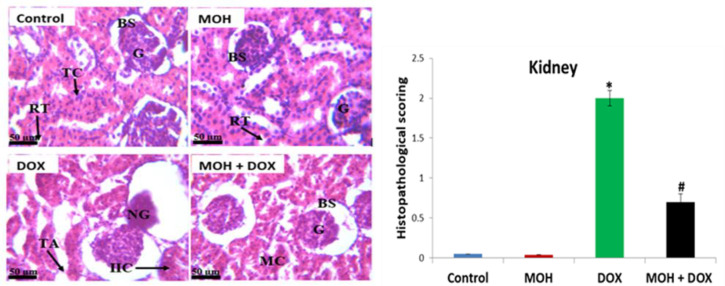
Photomicrographs of rat kidney administered with MOH and DOX (H & E stain, 400×, n=3). Control group: Bowman space (BS), glomerulus (G), renal tubule (RT); MOH group: Bowman space (BS), glomerulus (G), renal tubule (RT); DOX group: Necrotic glomerulus (NG), Infiltrated inflammatory cells (IIC), tubular atrophy (TA); MOH + DOX: Mild clumping of renal tubules (MC) (p<0.05). MOH: Morin hydrate; DOX: Doxorubicin. *Significant difference compared to the normal control (p<0.05); **#**significant difference compared to the DOX group

Figure 5 depicts the renal tissue sections from rats observed for histopathological changes. Group 1 and 2 (control and MOH) showed renal morphology with normal glomeruli (G) in Bowman’s capsule space (BS). The DOX group revealed tissue lesions consistent with necrotic glomerulus and tubular degeneration/atrophy and infiltrated inflammatory cells. However, the MOH+DOX group showed normal glomerulus and capsule space, although with mild to moderate clumping of renal tissue (H and E, x400).

## Discussion

Although pharmacological research is an increasing effort to detect drug targets to unravel action mechanism of drugs and to minimize side effects, the current toxic side effects of conventional anticancer drugs are worrisome (Wang et al., 2021 ▶). Doxorubicin (DOX) is a DNA interacting anticancer agent with a wide spectrum of clinical applications. Its toxicity on delicate organs, like the liver, heart and kidney, is a source of challenge for clinicians (Guo et al., 2016 ▶). We have evaluated, in this study, whether MOH could protect the liver and kidney from DOX toxicity. The molecular structures of the two compounds used in this study are indicated in Figure 1.

In this study, the acute injection of DOX induced hepatorenal damage demonstrated by notable increases in serum markers of AST, ALT, ALP, creatinine, uric acid and urea, with concomitant decreases in TP and ALB levels (Table 2). Increased serum activities of AST, ALT and ALP are known diagnostic markers of hepatocyte injury and biliary derangement. Also, renal damage causes kidney malfunction which was obvious herein via elevated serum urea and creatinine levels; and these markers are diagnostic indices of nephrotic damage and glomerular filtration rate reduction (Famurewa et al., 2018 ▶). The significant alterations in the serum markers indicate DOX-induced hepatotoxicity and nephrotoxicity as earlier reported by systematic experimental studies (Afsar et al., 2020 ▶; Benzer et al., 2018 ▶; Liu et al., 2021 ▶). The histology analyses depicted pathological alterations consistent with hepatorenal necrosis that might have weakened the cell membrane, leading to extracellular efflux of the enzymes and marked reduction of hepatic and renal functions (Figures 4 and 5). However, we observed that MOH administration prevented and restored the serum activities and levels of the altered markers. This observation indicates the protective potential of MOH against DOX-induced liver and kidney damage in this study. Our finding, therefore, agrees with earlier reports that MOH protects against cisplatin-induced kidney and liver toxicity (Athira et al., 2016 ▶; Singh et al., 2018 ▶).

The mechanism underlying DOX-mediated damage in healthy tissues is related to depression of antioxidant mechanism and consequent oxidative stress status (Kuzu et al., 2018 ▶; Prasanna et al., 2020 ▶). Therefore, our study considered endogenous enzymes and non-enzymes mediating cellular antioxidant mechanism and lipid peroxidation. Intriguingly, DOX injection to rats exerted significant depression on hepatorenal antioxidant status underscored by the prominent decreases in the activities of GPx, CAT, SOD and GSH levels as well as the pronounced increase in MDA level (Figures 2 and Figure 3). It is well known that cellular antioxidant homeostasis, deactivation and removal of ROS are mediated by GPx, SOD, CAT, and GSH. Superoxide dismutase is the key deactivator of superoxide radical through dismutation reaction to produce H_2_O_2_. CAT, a peroxisomal heamprotein, catabolizes H_2_O_2_ to H_2_O and molecular oxygen providing defense against ROS (Egba et al., 2022 ▶; Famurewa et al., 2020 ▶). The synergistic actions of GPx and GSH detoxify hydroperoxyl radicals and utilize the sulfhydryl group of GSH to protect lipids and protein targets of ROS to avert the course of lipid peroxidation. It is thus biochemically obvious that DOX-induced depression of SOD, CAT, GPx and GSH would promote lipid peroxidation as evident by significant elevation of MDA in this study as well as in the study of Kuzu et al. (2019) ▶. Our findings agree with reports in the existing literature that DOX is a trigger of antioxidant dysfunction and promoter of oxidative stress in off-target healthy organs (Hussain et al., 2021 ▶, Kuzu et al., 2018 ▶; Liu et al., 2021 ▶; Prasanna et al., 2020 ▶). Interestingly, MOH inhibited the deleterious action of DOX-induced oxidative stress in the liver and kidney. The hepatorenal antioxidant enzyme activity was markedly restored and the lipid peroxidation was mitigated by modulatory effect of MOH. 

Natural flavonoids are known to possess potent antioxidant efficacy and several other pharmacological activities (Moghaddam et al., 2020 ▶). MOH is a flavonoid with strong free radical scavenging capacity; it did not lower the anticancer efficacy of either DOX or mitomycin C during co-administration (Kok et al., 2000 ▶). Previous research data have delineated its antioxidant potency in stabilizing tissue antioxidant mechanism (Athira et al., 2016 ▶; Hassan et al., 2020 ▶; Ozdemir et al., 2022 ▶). For example, studies by Kuzu et al. (2018) ▶ and Ozdemir et al. (2022) ▶ show that MOH increases activities of GPx, SOD, CAT, and GSH levels and decreases MDA in the brain, liver and heart of rats injected with DOX and ifosfamide. Therefore, the antioxidant effect of MOH in this study may be associated with its ROS-scavenging potential within the liver and kidney, and this is consistent with Kuzu et al. (2019) ▶ study. 

Moreover, the DOX-induced oxidative stress milieu caused production of NO via activation of inducible nitric oxide synthase (iNOS). The crosstalk that exists between oxidative stress and pro-inflammation is well established in the literature (Donmez et al., 2020 ▶; Famurewa et al., 2019 ▶). The levels of NO in the DOX group were considerably increased in comparison to the normal control group levels in this study. Although possible upregulation of iNOS was not determined, its activation might generate overproduction of NO, indicating an oxidative stress-induced inflammatory response (Famurewa et al., 2019 ▶). The reaction of NO exerts inflammatory tissue damage, and concomitant reaction of NO with ROS could induce further damage. The finding here could be related to our histological observations of infiltration of inflammatory cells and necrosis. On the contrary, MOH inhibited the increase in NO levels. By implication, the NO production by the DOX-induced ROS in the liver and kidney was prevented by the MOH action. This underscores the antioxidant and anti-inflammatory effects of MOH. In the same vein, the histological abrasions in both organs were also alleviated and were comparable to the normal control group morphology.

In conclusion, DOX injection induced impairment in hepatorenal antioxidant mechanism which promoted lipid peroxidation and inflammatory overproduction of NO. Administration of MOH to the DOX-injected rats prevented hepatic and renal oxidative stress via potentiation of antioxidant homeostasis and reversal of NO overproduction in rats.

## Conflicts of interest

The authors have declared that there is no conflict of interest.
